# Sub-chronic toxicity determination of powdered *Tenebrio molitor* larvae as a novel food source

**DOI:** 10.1016/j.toxrep.2024.01.002

**Published:** 2024-01-09

**Authors:** Oleen Machona, Mirabel Mutanga, Farisai Chidzwondo, Rumbidzai Mangoyi

**Affiliations:** Department of Biotechnology and Biochemistry, University of Zimbabwe, Zimbabwe

**Keywords:** *Tenebrio molitor* larvae, Sub-chronic toxicity, Sprague- Dawley rats, Novel food source

## Abstract

*Tenebrio molitor* larvae are the first insect species to be given a favorable assessment by the European Food Safety Authority (EFSA) as a novel food source, enabling consumption of whole insect larvae or larvae that have been powdered and processed into a variety of food products. Pressure from economic hardships and increase in population growth have paved a way for the realization of an alternative food source in Zimbabwe. This study focused on determining the potential toxicity of *Tenebrio molitor* larvae powder as an alternative food source for humans. To determine the sub-chronic toxicity of *Tenebrio molitor*, the powder was administered daily by oral gavage to Sprague-Dawley rats at dose levels of 0, 300, 1000 and 3000 mg/kg for 70 days. A toxicological assessment which included mortality, appearance of clinical symptoms, food consumption, organ and body weight changes were performed. There were no treatment-related mortalities, clinical signs, changes in food consumption, body and organ weights observed during the treatment period. The study's findings suggest *Tenebrio molitor* larvae to be a good alternative as it did not appear to affect the rats' normal physiological and metabolic processes hence can be considered safe for human consumption. However, further studies on hematological, histological and biochemical markers may be necessary for confirmation of these current results.

## Highlights

• *Tenebrio molitor* larvae being reared in Zimbabwe as a novel food source are safe to consume.
• *Tenebrio molitor* larvae have no effects on liver enzymes upon long term exposure.
• *Tenebrio molitor* larvae can be commercialized.

## Graphical Abstract

**Figure:**
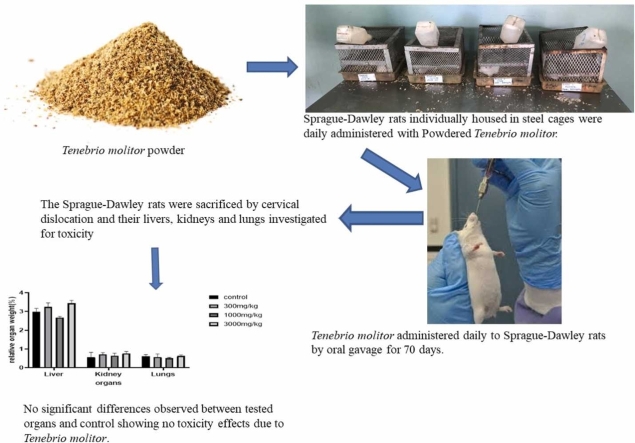

## 1 Introduction

Since ancient times, consumption of insects as a food source (entomophagy) has been a widespread habit in many countries. An estimate of around two billion people across Africa, Australia, Asia, Central and South America has been reported to currently consume insects because of their good taste, nutrient rich and are a cheap food source [10]. Thus, as the world's population is increasing and so is the demand for food, edible insects have the potential to become a significant global future food source. With their high feed conversion efficiencies, low environmental impact in terms of greenhouse gas emissions and yield of production in terms of the amount of land needed to produce one kilogram of protein from insects, edible insects unquestionably offer a beneficial answer to both current and future food insecurity [2].

One such insect which has been identified as a promising source of food and feed due to its high protein content, low environmental impact, and ease of cultivation is the mealworm *Tenebrio molitor*. *Tenebrio molitor* is believed to have originated from Europe but have spread globally and is being consumed at the larval stage in many parts of the world including Asia, Africa and South America [13]. Studies have shown that *Tenebrio molitor* larvae are a good source of nutrients that can be used as food for humans, a source of animal feed, or as a dietary supplement as they have the advantage of being easy to rear, having a short life cycle and high protein content, and thus can be produced in large quantities for these purposes.

*Tenebrio molitor* has also been authorized by the Commission Regulation (EU) 2017/893 as feed for monogastric animals such as poultry and swine and in aquaculture systems [15]. *Tenebrio molitor* larvae are also the first insect species to receive a positive evaluation from the European Food Safety Authority (EFSA) as a novel food, allowing the consumption of whole insect larvae or powdered larvae in various food products. In May 2021, the European Commission adopted regulations permitting the sale of dried mealworms in the European market. According to the EFSA’s evaluation all population groups could consume mealworms as whole insect larvae that have been thermally dried (blanched or oven-dried), ground into a powder, or added to a variety of food products like snacks, pasta, and biscuits.

However, as with other animal or plant-based diets, it is important to consider the possible risks associated with consuming edible insects as food. Consumption of mealworms may be linked to a number of risk factors for human health, including allergies, contamination with pathogenic microorganisms, pesticide residues, accumulation of heavy metals, parasites and harmful toxins. Despite having a number of studies reported in literature, not enough information is available on the effects of *Tenebrio molitor* on liver enzymes of the consumers. The liver plays a crucial role in determining toxicity due to its role in metabolism, transportation and removal of foreign substances from the body [16]. However, the liver's ability to metabolize drugs can be influenced by various factors such as inflammation and oxidative stress, which could potentially inhibit drug-metabolizing enzymes. Thus, this current study focusses on a comprehensive sub-chronic toxicity study to evaluate the safety of *Tenebrio molitor* larvae being reared in Zimbabwe as a novel food source, putting into account their effects on liver enzymes upon long term exposure. This study will provide valuable information on the safety of *Tenebrio molitor* larvae as a novel food source, which will be crucial for its safe consumption and commercialization.

## 2 Materials and methods

### 2.1 Preparation of *Tenebrio molitor* larvae powder

*Tenebrio molitor* larvae (mealworms) were obtained from the Natural Science and Technology Research (NSTR) laboratory where they were bred and were processed into a powder. Based on their color and morphology the larvae were identified as *Tenebrio molitor Linnaeus*. Upon removing the larvae from the containers in which they were bred, they were washed three times in cold tap water to remove debris and impurities. The mealworms were then blanched in hot water for three minutes and excess water was removed from the blanched mealworms. A paper towel was used to dry the mealworms then they were oven dried at 60 ºC for 12 h. Once dry they were ground into a fine powder using a blender and were sieved to obtain uniform particle size.

### 2.2 Experimental animals

**Fig. 1:**
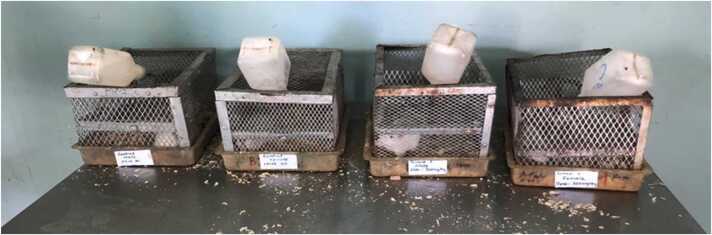
Sprague-Dawley rats individually housed in steel cages.

Eight healthy Sprague-Dawley (SD) rats weighing (50–65 g) were purchased from the Animal house of the University of Zimbabwe. The rats were housed in a temperature-controlled room (23–25 ºC) with a 12 h light-dark cycle (0600–1800). The rats were acclimatized to the housing conditions for 7 days prior to the oral administration of the *Tenebrio molitor* larvae powder. The animals were assigned randomly to four groups, a control and three treatment groups each consisting of one male and one female rat. The rats were individually housed in steel cages with bedding made of wood shavings which were changed when necessary (Fig. 1). Clean tap water and pellet food (National foods (PVT), Ltd., Harare, Zimbabwe) were provided *ad libitium*. The behavior of the rats was observed daily during the acclimation period.

### 2.3 Ethical considerations

The rats were handled with care ensuring their welfare and minimizing stress and in accordance with the guidelines provided [9].

### 2.4 Sub-chronic toxicity determination

Powdered *Tenebrio molitor* larvae was administered daily to Sprague-Dawley rats by oral gavage for 70 days. Olive oil was used as the vehicle and was administered to the control group so that rats received an equivalent volume to those given the powder. Both rats received a standard diet of pellets and the 3 treatment groups received the standard diet supplemented with powdered *Tenebrio molitor* larvae. The powder was administered at doses of 0, 300, 1000 and 3000 mg/kg. These doses were chosen such that the doses previously used in other studies are incorporated [7], which were reported to have not resulted in any adverse effects or toxicity. This allowed for the inference of dose-dependent parameters.

The powder was suspended in olive oil at an appropriate concentration for each dose. SD rats were weighed on the first day of acclimatization and beginning of treatment then once weekly thereafter. The initial body weight served as a basis for the dosing volume of 10 ml/kg used throughout the study period. This was adjusted weekly to sustain the target dose for the rats. The rats were monitored daily for signs of toxicity, behavioral changes and mortality. Food consumption was recorded once a week and the food consumed by each rat was calculated as g/rat/day. Food consumption was determined by weighing the amount of food (pellets) given to each rat then weighing the remaining food after 24 hrs.

After the treatment period the animals were fasted overnight before sacrificing by cervical dislocation. Rat livers were perfused in situ with ice cold normal saline (0.9 % w/v sodium chloride) using a Pharmacia LKB pump P-1 (Pharmacia, Uppsala, Sweden) until pale. This was done in order to remove excess blood from the livers. The livers were removed and bloated onto a dry tissue paper and weighed. The relative organ weight for the livers, kidneys and lungs were determined as a percentage of terminal body weight using the following formular

$$\mathrm{relative}\;\mathrm{organ}\;\mathrm{weight}\left(\%\right)=\frac{\mathrm{absolute}\;\mathrm{weight}\;\mathrm{of}\;\mathrm{organ}(g)}{\mathrm{body}\;\mathrm{weight}\;\mathrm{of}\;\mathrm{animal}}\times 100$$

Bilateral organ weights were determined by weighing the left and right organs separately and averaging the results. The livers were placed each in beakers containing a 1:5 0,1 M Tris-HCL buffer (pH 7.4) in preparation of protein determination.

### 2.5 Preparation of microsomal fractions from rat livers

Tissue fractions were kept cold and temperature maintained at 0- 4 ºC through-out the whole procedure. This was done by using pre-cooled homogenization medium, keeping beaker's centrifuge rotors and tubes in ice. The livers in Tris-HCL buffer were cut using scissors into smaller pieces and homogenized using mortar and pestle until a smooth homogenate was obtained. The liver homogenates were centrifuged at 10, 000 g (7500 rpm) for 30 min at 4 ºC using a Beckman Optima LE-80 k ultracentrifuge (Beckman Instruments Inc., California, USA). The supernatant was transferred into clean pre-cooled centrifuge tubes and centrifugation was done at 100,000 g (30,000 rpm) for 60 min at 4 ºC using a Beckman Optima LE-80 k ultracentrifuge (Beckman Instruments Inc., California, USA) to obtain the microsomal pellet and supernatant fraction (cytosol). The supernatant was aliquoted into labeled 1.5 ml microtubes and stored at –80 ºC in preparation of protein determination.

### 2.6 Protein concentration determination- Lowry assay method

Protein content in the rat liver cytosolic fractions was determined by the Lowry assay. Tris- HCL buffer was used to dilute the supernatant into X50 and X200 dilutions and a volume of 250 µl of each of the protein dilutions was added to test tubes with the same volume of 0.5 M NaOH. This was done in duplicate for each sample dilution and was immediately vortexed.

Two reagent blanks were prepared by adding 500 µl of NaOH in tubes and BSA standards were prepared in duplicate [11]. A volume of 2.5 ml of freshly prepared Lowry reagent was added to all the tubes including the standards and after 10 min of incubation, the same volume of Folin and Ciocalteu’s phenol reagent was added to the tubes.

Absorbance was read at 720 nm wavelength after incubating for 30 min using a Shimadzu UV 1601 spectrophotometer.

### 2.7 Statistical analysis

Statistical analysis of the results was carried out using One-way Analysis of variance (ANOVA) followed by multiple comparisons with Dunnett’s test to determine which mean values were significantly different from the control values. All computations were done using Graph Pad Prism software (Version 9.5.0, Graph pad Software Inc, San Diego, USA). Significant differences were considered at p < 0.05 and all the results were expressed as the mean ± standard deviation of the mean.

## 3 Results

### 3.1 Oral sub-chronic toxicity test

During the 70-day study period, daily administration of powdered T*enebrio molitor* larvae to Sprague-Dawley rats resulted in no mortality of the rats. No clinical symptoms were observed during acclimatization and the 70-day treatment period. Clinical signs which were being observed included changes in behavior, skin, eyes and fur colour.

### 3.2 Body weight changes

**Fig. 2:**
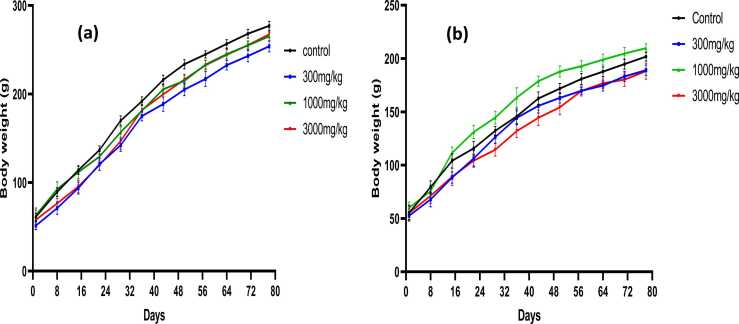
Changes in body weight **(a)** of male SD rats and **(b)** of female SD rats fed with powdered *Tenebrio molitor* larvae recorded weekly from the first day of acclimatization to the day of necropsy. Body weight increased as the study progressed. P < 0.001.

The mean body weights of SD rats administered with powdered *Tenebrio molitor* larvae powder at different dosages as well as the control group for a period of 70 days is graphically illustrated in Fig. 2. Body weight measurements were used to evaluate the health status of the animals during the treatment period. Results shows that for all the concentrations tested, rats’ body weight increased, showing that T*enebrio molitor* larvae powder has no effects on growth of the rats. There was no significant difference among the body weights of all the rats fed with *Tenebrio molitor* and the control rat, with P < 0.001.

### 3.3 Food consumption

Amount of food consumed by each rat was determined once a week by weighing the amount of food (pellets) given to each rat then weighing the remaining food after 24 h.

**Fig. 3:**
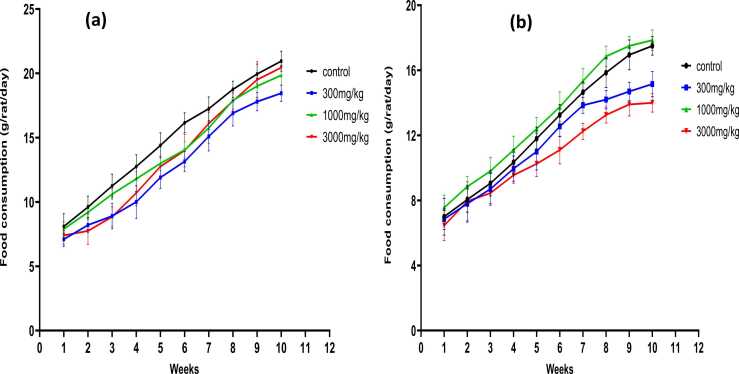
**:** Effects of powdered *Tenebrio molitor* larvae on food intake in male and female SD rats recorded once weekly during the treatment period of 70 days. **(a)** average food consumed by male rats **(b)** average food consumed by female rats. A gradual increase in terms of food intake as the number of days increased is observed.

Food consumed was expressed as food consumed in grams per rat per day and the data collected was used to come up with the graphs shown in Fig. 3. Results show that *Tenebrio molitor* larvae does not lead to loss of appetite in the rats hence the normal behavior and feeding patterns of the rats were not altered. There was no significant differences among the food consumed by all the male rats fed with *Tenebrio molitor* and the control rat with p value of < 0.001. However, though all the rats did not lose appetite, there was a slight difference of p = 0.055 for the females fed with 300 and 3000 mg/kg of *Tenebrio molitor*.

### 3.4 Relative organ weight

**Fig. 4:**
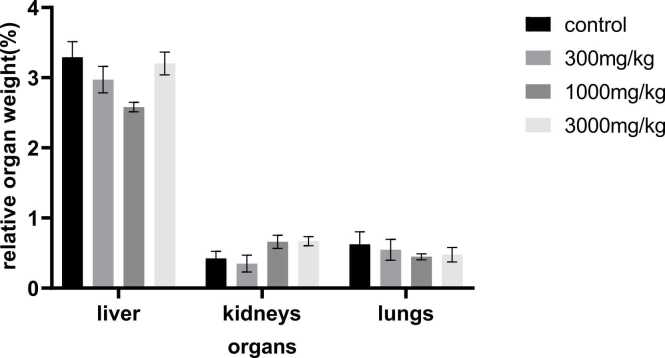
**:** Effects of powdered *Tenebrio molitor* larvae on relative organ weight of male Sprague Dawley rats. Relative organ weight was expressed as a percentage of terminal body weight of the rat.

**Fig. 5:**
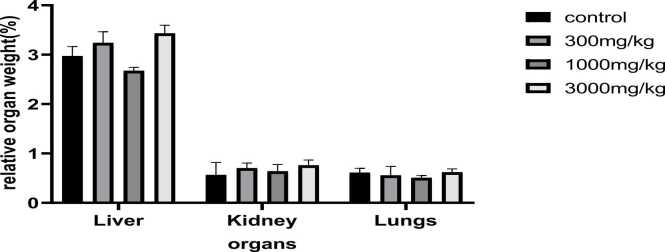
**:** Effects of powdered *Tenebrio molitor* larvae on relative organ weight of female Sprague Dawley rats. Relative organ weight was calculated as a percentage of terminal body weight of the rat.

The rats were sacrificed after the 70-day treatment period and the liver, kidney and lungs were quickly removed and weighed. Before the animals were sacrificed their body weights were recorded and these were further used to calculate the relative organ weights (Fig. 4, Fig. 5). For both male and female groups, no significant differences were observed among all organs weight and the controls, hence no effect due to *Tenebrio molitor* at all concentrations. The p value was found to be <0.001. The results show that the liver has a comparatively larger weight when compared to the kidney and lungs as expected since the liver is the largest internal organ.

### 3.5 Protein determination

The liver cytosol Protein content was determined using the Lowry assay, and Bovine Serum Albumin (BSA) was used as the reference standard. A BSA standard curve was generated from the prepared standards and the protein concentration was determined by interpolating the obtained absorbance values of the protein samples from the BSA standard curve. The concentration obtained from the graph was in µl/ml and the concentrations were converted to mg/ml as follows: mg/ml = µl/ml/1000 x dilution (500/250) x dilution factor carried out.

**Fig. 6:**
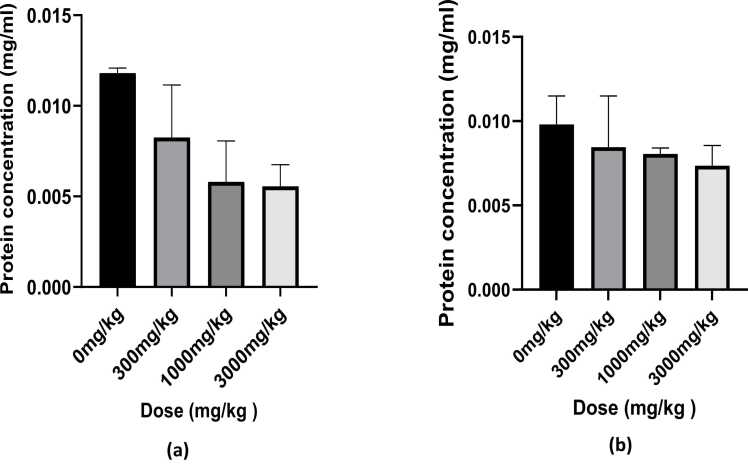
The effect of different doses of *Tenebrio molitor* larvae powder on liver protein content. No significant difference was observed for both sexes **(a)** liver protein content in female rats **(b)** liver protein content in male rats.

The results show no significance differences when compared to the control with p < 0.001. Graphs were generated in graph pad prism using the interpolated values and the graphs are depicted in Fig. 6.

## 4 Discussion

The larval form of *Tenebrio molitor* which is also known as mealworm, has been proposed as a novel source of food as it has been found to be highly nutritious and more beneficial to humans as compared to conventional meat sources. In Zimbabwe many edible insects are consumed as food but however introduction of new insect species such as *Tenebrio molitor* gives rise to food safety issues. Human health is critically dependent on food safety, particularly when it comes to new sources of food. Consumption of edible insects poses ongoing food safety risks such as the possibility of the insect being toxic or it may have picked up pathogens or microorganisms during its life cycle, it can get spoiled especially after harvesting, and it may also cause allergies in certain individuals [14]. This current study focused on determining the possible toxic effects of *Tenebrio molitor* larvae in its powdered form as a novel food source for humans. Evaluations were carried out in Sprague-Dawley rats over a period of 70 days.

Over the course of the study, no treatment related mortalities were observed. During clinical observations, there were no clinical changes observed in terms of changes in skin, fur and eye color as well as change in behavior. Clinical signs can be in a form of change in food eating habits, change in physical appearance or in some instances diarrhea. According to [8], diarrhea is a common observation in toxicity studies and can be as a result of stress, changes in diet or as a direct toxic effect on the gastrointestinal tract caused by the test substance. However no clinical signs were observed in this study and this is consistent with the results obtained by Han et al., [6], [7], in which he observed no clinical symptoms after administering freeze dried *Tenebrio molitor* larvae powder for 28 and 90 days respectively.

Administration of *Tenebrio molitor* did not have any effect on the body weights in both sexes. Changes in body weight are an important endpoint in toxicity studies that might offer preliminary information about the test substance [17]. All the rats used in the study gained weight during the treatment period thus indicating that the powder did not alter or prevent weight gain in rats. Comparison between male and female rats showed that male rats gained more weight as compared to females and this has been observed in many toxicity studies and Maric et al., [12] reports that males generally gain more weight than females. This is because males have a higher predisposition towards weight gain due to differences in the expression of genes related to metabolism and fat storage.

A One-way ANOVA was performed to determine whether there were significant differences between the mean response of the control group and the treatment groups. The results for both male and female rats showed a statistically insignificant difference (p > 0.05) between the control group and the treated groups. These results are similar to those observed by [6] who evaluated the safety of freeze- dried powdered *Tenebrio molitor* larvae as a novel food source. He also observed no significant differences in food consumption in both male and female rats. This correlates with the observations in this current study in which differences in food consumption between the control and the treatment groups was insignificant (p > 0.05).

A comparison between male and female rats in terms of food consumption shows that male rats consumed more than females. This coincides with the results from body weight changes as it is expected that the more weight the rat has, the more likely it is to consume more food.

According to Asarian and Geary, [3], male rats' total daily calorie consumption exceeds that of female rats by a greater amount than would be expected given their larger lean body mass and metabolic rate. Nevertheless, results from this study show that powdered *Tenebrio molitor* larvae did not seem to alter the rats’ healthy physiological and metabolic functions as it had no detrimental effects on the rat’s development or suppression of appetite.

In both humans and animals, relative organ weight can serve as an indicator of pathological and physiological condition. Primary organs such as the heart, liver, kidney, lung and spleen are mostly affected by metabolic reactions induced by toxic chemicals. Changes in organ weight can be a sign of impairments in the normal body functioning. The ratio of organ weight to body weight can reveal organ enlargement, atrophy, or hypertrophy [17].

After administering the test material for 70 days, an autopsy was conducted, and the absolute weight in grams and relative (%) weights of the organs were recorded. In this current study the relative organ weight for the liver, kidneys and lungs was determined. The results show that the liver has a larger weight than the kidneys and lungs since the liver is the largest internal organ [1]. However, there were no appreciable differences (p > 0.05) in the relative organ weights percentages between the weights of the control groups and that of the treatment groups in both sexes.

These results correspond to those of in-vivo experiments carried out by [5], in which the toxicity of powdered *Tenebrio molitor* larvae ingested with expanded- polystyrene were investigated.

A number of studies have been carried out to investigate the potential toxicity of *Tenebrio molitor* larvae powder as a novel food source [5], [6], [7]. However, these studies did not investigate the total liver protein quantity in order to determine if the powder poses any effect on the liver. The liver is the major organ involved in the metabolism of foreign substances in the body. However, the majority of organs have quantifiable amounts of the P450 enzymes for the oxidation and reduction pathways, which catalyze the reactions involved in biotransformation. It has been demonstrated that organ-specific activation by P450 is significant in the lung and the olfactory tissue and is the cause of organ toxicity for numerous substances [1].

Liver protein content was determined using the Lowry method of protein determination. In male rats, the control group was observed to have higher protein content as compared to the other treatment groups although the differences were statistically insignificant (p > 0.05). A decrease in protein concentration in the liver can be due to a toxic substance that directly damages the liver and affect its ability to produce proteins [4]. However, since all the other parameters determined in this study did not suggest *Tenebrio molitor* to be toxic and the fact that the differences were insignificant it can thus be concluded that the powder had no effect on the liver.

## 5 Conclusion

Administration of powdered *Tenebrio molitor* larvae to Sprague-Dawley rats for a period of 70 days did not result in adverse effects in terms of altering the rats’ intake of food, growth and health status of the liver. The results were comparable to those in literature and thus suggesting *Tenebrio molitor* larvae to be a good source of protein for people.

## CRediT authorship contribution statement

**Mangoyi Rumbidzai:** Supervision, Writing – original draft, Writing – review & editing. **Chidzwondo Farisai:** Validation, Writing – review & editing. **Mutanga Mirabel:** Investigation. **Machona Oleen:** Investigation, Validation.

## Declaration of Competing Interest

The authors declare that they have no known competing financial interests or personal relationships that could have appeared to influence the work reported in this paper.

## Data Availability

No data was used for the research described in the article.
